# The Role of Oxidative Stress in the Development of Systemic Sclerosis Related Vasculopathy

**DOI:** 10.3389/fphys.2018.01177

**Published:** 2018-08-24

**Authors:** Amaal E. Abdulle, Gilles F. H. Diercks, Martin Feelisch, Douwe J. Mulder, Harry van Goor

**Affiliations:** ^1^Department of Internal Medicine, Division of Vascular Medicine, University Medical Centre Groningen, University of Groningen, Groningen, Netherlands; ^2^Section Pathology, Department of Pathology and Medical Biology, University Medical Centre Groningen, University of Groningen, Groningen, Netherlands; ^3^Clinical and Experimental Sciences, Faculty of Medicine, University of Southampton, Southampton, United Kingdom

**Keywords:** systemic sclerosis, vasculopathy, reactive oxygen species, development, biomarker, intervention

## Abstract

Systemic sclerosis (SSc) is a rare connective tissue disease characterized by autoimmunity, vasculopathy, and progressive fibrosis typically affecting multiple organs including the skin. SSc often is a lethal disorder, because effective disease-modifying treatment still remains unavailable. Vasculopathy with endothelial dysfunction, perivascular infiltration of mononuclear cells, vascular wall remodeling and rarefaction of capillaries is the hallmark of the disease. Most patients present with vasospastic attacks of the digital arteries referred to as ‘Raynaud’s phenomenon,’ which is often an indication of an underlying widespread vasculopathy. Although autoimmune responses and inflammation are both found to play an important role in the pathogenesis of this vasculopathy, no definite initiating factors have been identified. Recently, several studies have underlined the potential role of oxidative stress in the pathogenesis of SSc vasculopathy thereby proposing a new aspect in the pathogenesis of this disease. For instance, circulating levels of reactive oxygen species (ROS) related markers have been found to correlate with SSc vasculopathy, the formation of fibrosis and the production of autoantibodies. Excess ROS formation is well-known to lead to endothelial cell (EC) injury and vascular complications. Collectively, these findings suggest a potential role of ROS in the initiation and progression of SSc vasculopathy. In this review, we present the background of oxidative stress related processes (e.g., EC injury, autoimmunity, inflammation, and vascular wall remodeling) that may contribute to SSc vasculopathy. Finally, we describe the use of oxidative stress related read-outs as clinical biomarkers of disease activity and evaluate potential anti-oxidative strategies in SSc.

## Systemic Sclerosis

Systemic sclerosis (SSc) is a complex chronic autoimmune disease, characterized by vasculopathy, low-grade chronic inflammation, and fibrosis of the skin and internal organs; the latter causes organ dysfunction, potentially leading to severe morbidity and premature mortality. Vasculopathy is the central feature of the majority of SSc-related complications, for which treatment options are very limited; this has led to a largely unmet medical need. Globally, the incidence rate of SSc appears to have gradually increased over recent decades, with approximately 7–20 million individuals being diagnosed annually (Nikpour et al., 2010). This increase may be due, in part, to improved diagnosis, but it may also be linked to environmental, nutritional, or other lifestyle-related factors that contribute to the surge in cardiometabolic diseases over the same time span. Systemic sclerosis affects women more frequently than men, and previous reports indicate a slightly higher susceptibility to the disease among African Americans as compared to Caucasians (Laing et al., 1997; Mayes et al., 2003; Ranque and Mouthon, 2010). The two known subtypes (i.e., limited cutaneous SSc and diffuse cutaneous SSc) differ in course and prognosis. The diffuse cutaneous subtype is often associated with the manifestation of devastating complications early in the course of the disease, while such complications occur more insidiously in the limited cutaneous subtype. Although auto-immunity, inflammation, and oxidative stress have all been implicated in the pathogenesis of vasculopathy, the definite factors that initiate this disease remain un-identified (Gabrielli et al., 2009).

**FIGURE 1:**
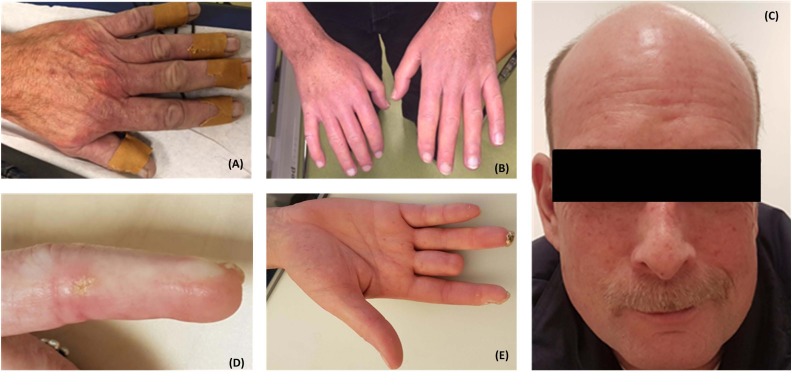
Patients with systemic sclerosis (SSc) often present with Raynaud’s phenomenon **(A)**. Patients may subsequently develop tightening and thickening of the skin of the fingers (sclerodactyly) **(B)**, telangiectasia **(C)**, calcinosis cutis **(D)**, and digital necrosis which may lead to amputation of the digits **(E)**. Written informed consent was obtained from the patients for the publication of these images.

Vasculopathy, which affects both small and large blood vessels, is characterized by endothelial dysfunction, perivascular infiltration of mononuclear cells, extracellular matrix remodeling in the vascular wall, and a loss of capillaries (capillary rarefaction) (Fleming et al., 2009; Mostmans et al., 2017). Raynaud’s phenomenon (RP) (**Figure 1**), which manifests in the form of recurrent vasospastic attacks that cause episodic discoloration of the extremities in response to cold or emotional stress, is the hallmark of SSc and generally the first detectable sign of the disease (LeRoy and Medsger, 2001; Herrick, 2005; Gabrielli et al., 2009). Following RP, patients may present with critical ischemia and ulceration, which can result in increased morbidity and mortality, as well as a decreased quality of life (Almeida et al., 2015). The SSc-specific abnormalities that occur in the capillaries (e.g., dilatation of capillaries in early stages and loss in later phases) can easily and non-invasively be assessed using nailfold capillaroscopy, which is routinely used in the clinical setting.

**FIGURE 2:**
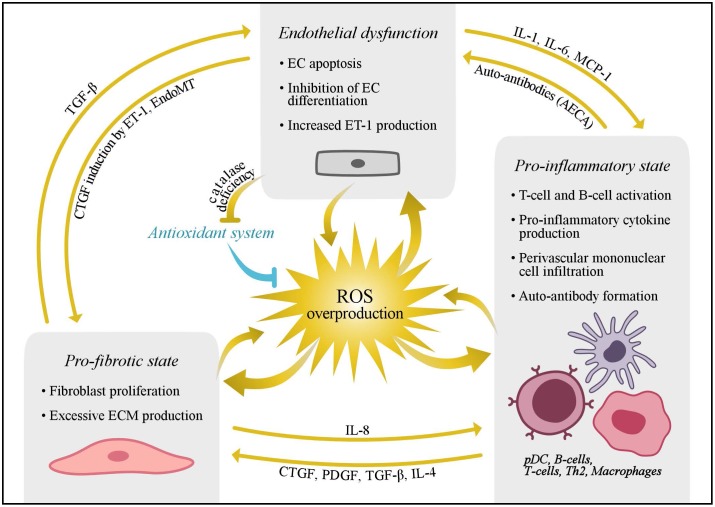
Schematic presentation of the key elements in the pathogenesis of SSc. Overproduction of reactive oxygen species may induce endothelial dysfunction and promote a pro-fibrotic and pro-inflammatory state. Moreover, interplay between these processes has been proposed to further cause damage, and various cytokines and chemokines are thought to play an important role in this process. AECAs, anti-endothelial cell antibodies; CTGF, connective tissue growth factor; ECs, endothelial cells; EndoMT, endothelial to mesenchymal transformation; ECM, extracellular matrix; ET-1, endothelin-1; IL, interleukin; MCP, monocyte chemotactic protein 1; pDC, plasmacytoid dendritic cells; PDGF, platelet derived growth factor; ROS, reactive oxygen species; TGF-β, transforming growth factor beta; Th, T helper cell.

There is abundant evidence in the literature that elevated production of reactive oxygen species (ROS)— a collective term for oxygen-derived species with enhanced chemical reactivity— or an unfavorable shift in oxidative/reductive tone in favor of a pro-oxidative milieu (a condition known as “oxidative stress”) is involved in a variety of cardiovascular risk factors and diseases (e.g., hypertension, atherosclerosis, and heart failure) (Laursen et al., 1997; Nakamura et al., 1998; Heinloth et al., 2000; Barry-Lane et al., 2001; Hirotani et al., 2002; Tocchetti et al., 2011; Wu et al., 2014). However, little is known about the role played by oxidative stress in the development of SSc-related vasculopathy. Following Murrell’s hypothesis (Murrell, 1993) that the pathogenesis of SSc is linked to the generation of ROS, several studies have presented evidence of abnormal ROS production in SSc which, if unabated, may subsequently cause damage (**Figure 2**). Although chronically elevated levels of ROS have been suspected to prolong disease, oxidative stress may also be an initiating factor in the development of SSc vasculopathy. Therefore, a better understanding of the role played by oxidative stress in the development of SSc vasculopathy could help to identify early interventions aimed at delaying the onset of overt symptoms and/or attenuating the course of the disease. In this review, we first describe the physiological role played by ROS in the vascular system. Thereafter, we attempt to explain the role of oxidative stress in the pathogenesis of SSc vasculopathy. Finally, we evaluate the potential of free radical-scavenging therapeutic interventions.

## The Role of Ros in Normal Vascular Physiology

### Production of Reactive Oxygen Species

**FIGURE 3:**
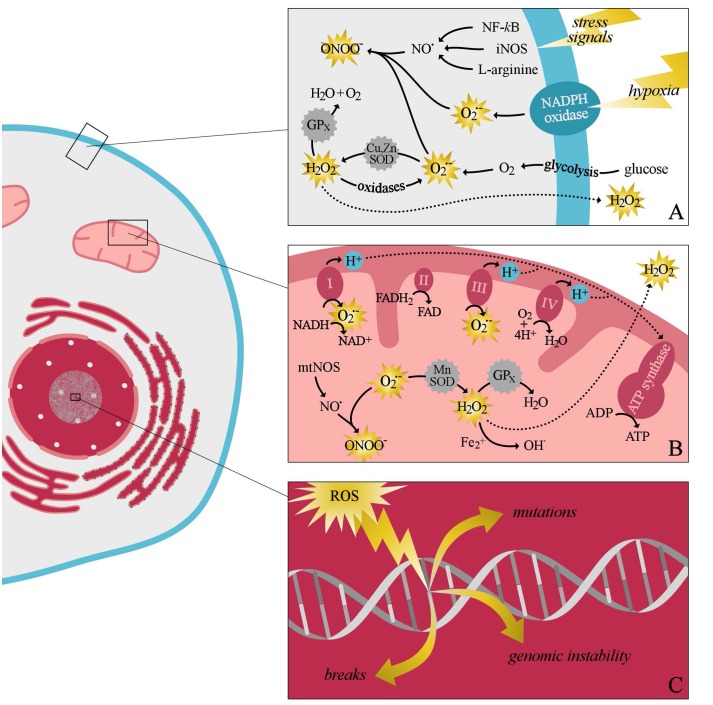
A simplified schematic overview of intracellular production of reactive oxygen species (ROS) and the intracellular antioxidant systems. Cytosolic ROS production can be evoked as a response to several stress signals, including hypoxia **(A)**. During this process superoxide anion is mainly produced by oxidases, including NADPH oxidase, xanthine oxidase, and lipooxygenase. After its production, superoxide is rapidly converted by the antioxidant system to, the less damaging radical, hydrogen peroxide and water. Furthermore, cytosolic ROS production can promote mitochondrial ROS production **(B)**. Superoxide anion produced by the different complexes of the electron transport chain can easily react with nitric oxide to form peroxy nitrite species. Alternatively, superoxide can also be converted by manganese superoxide dismutase (SOD) to hydrogen peroxide. Hydrogen peroxide can be either converted to water by glutathione peroxidase, or diffused to the cytosol. If the production of free radicals exceeds the antioxidants buffering capacity, damage to all component of the cell may occur **(C)**. ATP, adenosine triphosphate; ADP, adenosine diphosphate; FAD/FADH_2_, flavin adenine dinucleotide; Fe^2+^, iron; H_2_O_2_, hydrogen peroxide; H_2_O, water; NF-κB, nuclear factor kappa-light-chain-enhancer of activated B cells; iNOS, inducible nitric oxide synthase; NAD^+^/NADH, nicotinamide adenine dinucleotide; NO^•^, nitric oxide; mnSOD, manganese superoxide dismutase; mtNOS, mitochondrial nitric oxide synthase; ${\text{O}}_{2}^{•-}$, superoxide; OH^•^, hydroxyl radical; ONOO^−^, peroxynitrite; Cu,ZnSOD, copper- and zinc-containing superoxide dismutase; GP_x_, glutathione peroxidase.

Reactive oxygen species were first described by Fenton (1894) and they include free-radical species derived from oxygen such as superoxide anion (${\text{O}}_{2}^{•-}$) and hydroxyl radical (OH^•^) and also non-radical oxidants such as hydrogen peroxide (H_2_O_2_) (Murrell, 1993; Cumpstey and Feelisch, 2017). Reactive oxygen species can be formed non-enzymatically in reactions catalyzed by metals (as in the Fenton reaction); however, in biology, they are mainly generated through intracellular enzymatic sources, and all vascular cell types (including endothelial cells [ECs], smooth muscle cells, and fibroblasts) are able to produce ROS. The majority of ROS are generated during the production of ATP from molecular oxygen, a process also known as mitochondrial respiration (Balaban et al., 2005). During this process, dysregulated mitochondria produce excessive amounts of ROS which can damage all cellular components (Bolisetty and Jaimes, 2013). Mitochondrial electron transport chains consist of four inner-membrane complexes, with the majority of mitochondrial ROS produced by complexes I and III (**Figure 3**) (Berg et al., 2002). Superoxide anion produced by these complexes can be rapidly converted to H_2_O_2_ by superoxide dismutase (SOD) family of enzymes, comprised of manganese superoxide dismutase (MnSOD) and copper- and zinc- containing superoxide dismutase (Cu,ZnSOD) (Miriyala et al., 2012). Hydrogen peroxide is then converted to water by glutathione peroxidase, or diffused into the cytosol and, thereby, reduces the damaging effect of ROS to the mitochondria (Maharjan et al., 2014). Once ROS is diffused to the cytosol, reactive species are eliminated by the cytosolic antioxidant systems until the cytosolic redox buffer capacity is reached (Maharjan et al., 2014). Also, increased mitochondrial peroxynitrite formation may lead to decreased breakdown of ROS, due to the nitration and inactivation of MnSOD (Daiber, 2010). Furthermore, cytosolic ROS production by NADPH oxidases may trigger mitochondrial ROS formation through several processes. For instance, the opening of the redox sensitive K_ATP_ channels may lead to changes in the mitochondrial membrane potential, and thereby causing a rise in mitochondrial ROS production (Garlid et al., 2003).

Other sources of intracellular ROS production include nicotinamide adenine dinucleotide phosphate (NADPH) oxidase (NOX) enzymes (Bedard and Krause, 2007) uncoupled nitric oxide synthases (NOSs) (Landmesser et al., 2003; Taniyama and Griendling, 2003; Turrens, 2003; Kawashima et al., 2007; Nishino et al., 2008) xanthine oxidases and lipoxygenases (Murrell, 1993). In most of these reactions, ROS formation is a by-product of oxidative metabolism (and is often considered an undesirable result of electron leakage), although this is clearly not the case for NOX enzymes, whose sole purpose is the production of superoxide for signaling purposes or (at much higher rates of production) microbial killing. Mammals are equipped with antioxidant systems that offer protection against the damaging off-target effects of ROS, and several antioxidants and antioxidant enzymes have been described. The antioxidant buffering systems include low-molecular weight substances such as nutritional antioxidants (e.g., vitamin C and vitamin E) (Benfeito et al., 2013) sulfhydryl (SH)-containing compounds (e.g., thiols such as glutathione) (Turell et al., 2013) and antioxidant enzymes (including SOD, catalase, and glutathione peroxidases) (Matés et al., 1999). An imbalance between the production of ROS and their inactivation by antioxidants and reducing enzymes that results in ROS overproduction is referred to as “oxidative stress” (Betteridge, 2000; Grygiel-Górniak and Puszczewicz, 2014). In response to an increase in endogenous production of ROS, an organism may adapt by increasing its antioxidant capacity (commonly referred to as the redox-buffering capacity). If an organism is unable to maintain the appropriate redox homeostasis, disturbances in this balance can cause harmful effects that can ultimately damage the structural and functional components of a cell, leading to the oxidation of proteins, lipids, and DNA (Harman, 1956).

### Reactive Oxygen Species Signaling in Vascular Tone Regulation

Reactive oxygen species serve as important signaling molecules that regulate a variety of physiological processes in order to maintain appropriate redox homeostasis (Jones and Sies, 2015). In the vasculature, for example, ROS have been identified as key signaling molecules involved in the fundamental function of vascular smooth muscle cells (VSMCs), including the ability of blood vessels to contract or relax (MacKay and Knock, 2015). However, the available data underline the complexity of the effects of ROS on the vascular system, as —depending on the conditions— both contraction and relaxation of the vascular muscle may occur (Faraci, 2006). Superoxide anion has a contractile effect on VSMC, and removal of the endothelium prevents this vasoconstriction, making this process endothelium dependent (Katusic and Vanhoutte, 1989). In line with this finding, the toxicant benzo(a)pyrene has been found to decrease the endothelium-dependent NO-induced vasodilation in retinal arterioles through the production of superoxide (Kamiya et al., 2014). Vasoconstriction, as an acute response to ROS, is caused by the scavenging of endothelial nitric oxide (NO^•^), another free radical and a potent vasodilator produced in arterial blood vessels to regulate vascular tone and blood flow (Moncada et al., 1991). Chronically, vasoconstriction may also be caused by an increased expression of contractile proteins. Likewise, ROS production has previously been associated with a twofold increase in the expression of contractile proteins and microRNA (miR-145), which can up-regulate transcription of contractile protein genes (Chettimada et al., 2014). In addition, increased contractility of VSMCs by affecting the Ca^2+^ signaling pathway has previously been described (Touyz and Schiffrin, 2004). Several studies have also reported on the vasodilatory effect of ROS, especially in cerebral arteries. Superoxide, generated by xanthine and xanthine oxidase, produces dilation of cerebral arterioles, presumably mediated through potassium channel opening (Wei et al., 1996). Moreover, overproduction of H_2_O_2_ has been suggested to lead to vasodilatation through an enhanced endothelial response to acetylcholine (Drummond et al., 2000; Thomas et al., 2002; Zhou et al., 2013). A current hypothesis poses that ROS may have a biphasic response, with lower concentrations resulting in vasodilation and higher rates of production causing vasoconstriction (Faraci, 2006) potentially explaining the contradictory effects of ROS on the vascular system.

NO^•^, itself a free-radical reactive nitrogen species (RNS), is also suspected of playing a critical role in vascular homeostasis. NO^•^ is a potent activator of soluble guanylyl cyclase, which converts guanosine triphosphate (GTP) to the second messenger cyclic guanosine monophosphate (cGMP), causing vasodilation by increasing calcium handling, which stimulates the contractile function of the VSMC (Arnold et al., 1977). NO^•^ is synthesized from the amino acid L-arginine by a family of isoenzymes termed NOSs. The synthesis of NO^•^ occurs in response to various physicochemical stimuli, including neurotransmitters, shear stress, and growth factors. Furthermore, ${\text{O}}_{2}^{•-}$ and NO^•^ can readily interact with each other to form peroxynitrite (ONOO^−^); this reaction accounts for the vasoconstriction observed by enhanced ROS production and explains the vasorelaxant effects of SOD in experiments with isolated blood vessels. Prolonged exposure of ECs to peroxynitrite can lead to oxidation of the NOS cofactor, tetrahydrobiopterin, and protein tyrosine nitration, which may impair NOS function following the uncoupling of electron flow from arginine oxidation; these events ultimately translate into a low NO bioavailability and impaired vasodilatation (Aliev et al., 1998; Dimmeler and Zeiher, 2000). Hydrogen sulfide (H_2_S) — a reactive signaling molecule belonging to the group of “gasotransmitters” (Wang, 2002) and a reactive sulfur species (RSSs) (Cortese-Krott et al., 2017) produced by ECs and perivascular adipose tissue—may also promote vasodilation through the activation of endothelial NO synthase, the inhibition of cyclic guanosine monophosphate (cGMP) degradation, and by activating potassium channels in VSMCs (Bełtowski and Jamroz-Wiśniewska, 2014). Because of the propensity of ROS, NO, and H_2_S to chemically and functionally interact with each other at multiple levels, Cortese-Krott et al. (2017) have underscored the importance of viewing these reactive species as a unified entity (defined as the “reactive species interactome”), rather than as separate signaling entities.

In addition to the direct regulation of vascular tone, ROS are also essential for maintaining O_2_ hemostasis by stimulating several transcription factors, such as hypoxia-inducible factor-1 (HIF-1) (Chandel et al., 1998, 2000; Brocato et al., 2014; Friedman et al., 2014). Hypoxia increases ROS production by a variety of mechanisms, triggering marked alterations in redox signaling (Smith et al., 2017). Furthermore, ROS upregulate vascular endothelial growth factor (VEGF) expression via activation of HIF-1, subsequently leading to angiogenesis (Arbiser et al., 2002; Xia et al., 2007). Although these studies shed light on the possible role of ROS in O_2_ sensing, their exact role in these and other mechanisms often remains unclear and awaits further clarification. Of note, H_2_S is also suspected of being involved in O_2_ sensing (Olson, 2015) and the availability of both H_2_S and ROS is modulated by their interaction with NO (Cortese-Krott et al., 2017) as mentioned above.

### Reactive Oxygen Species Signaling in Other Cellular Processes and Immunity

Increasing evidence suggests that ROS also play an important role in a variety of cellular activities through several signaling pathways (e.g., the NF-κB signaling pathway and the MAPKs signaling pathway) (Zhang et al., 2016). Several experimental studies have demonstrated that a biphasic effect (i.e., low concentrations increasing the cell number and high concentrations decreasing the cell number) of ROS occur in fibroblasts, cultured smooth muscle cells, cultured ECs, and immortalized lung epithelial cells (Burdon, 1995; Arnold et al., 2001; Day and Suzuki, 2005). Additionally, human embryonic stem cells have previously been found to engage in spontaneous differentiation when they were cultured under normoxic conditions (21% O_2_); however, reduction of the oxygen saturation to 1% O_2_ inhibits cell proliferation (Ezashi et al., 2005). Hypoxia-driven ROS production could partially explain the described observations. Furthermore, both the innate and adaptive immune systems are thought to be affected by ROS production. For example, it has been reported that ROS play a crucial role in T-cell activation, a process that is also associated with an increase in cell ROS production (Devadas et al., 2002). These findings highlight the importance of ROS in the regulation of several cellular processes.

## The Role of Oxidative Stress in SSc Vasculopathy

### Link Between Oxidative and Endothelial Cell Dysfunction/Injury

The EC is believed to be a main target for pathological processes in SSc (Mostmans et al., 2017) and ROS have been implicated as having an important role in this process. In SSc, oxidative stress may cause the activation and damage of ECs (Grygiel-Górniak and Puszczewicz, 2014) leading to EC apoptosis and impairment of cell–cell adhesion (Sgonc et al., 1996). This impairment ultimately increases vascular endothelial permeability, subsequently leading to alterations in EC signal transduction (Fleischmajer and Perlish, 1980). When damaged, vascular ECs produce increased levels of vasoconstrictive mediators (e.g., endothelin-1 [ET-1]) and show an impaired release of NO and prostacyclin (Freedman et al., 1999; Romero et al., 2000; Matucci Cerinic and Kahaleh, 2002). This imbalance in mediators, in addition to several other oxidative and non-oxidative pathways (e.g., the adrenergic mechanism), results in altered vascular tone in favor of vasoconstriction. Consistent with our hypothesis that oxidative stress plays a crucial role in the development of SSc vasculopathy, ECs from SSc patients were previously reported to have the ability to produce H_2_O_2_ (Servettaz et al., 2007) which may inhibit endothelial differentiation due to a reduced octamer-binding transcription factor 4 (Oct-4) expression (Xiao et al., 2014). Furthermore, endothelin-1 and angiotensin II (a bioactive peptide of the renin–angiotensin system), which are often elevated in SSc, may upregulate mitochondrial ROS generation through the activation of NADPH oxidase (Nozoe et al., 2008; Wosniak et al., 2009; Wen et al., 2012). In addition to this ability to produce ROS, ECs are also more prone to ROS injury due to a deficiency in catalase synthesis (Jornot and Junod, 1992). This deficiency may initiate a self-perpetuating cycle of recurrent RP attacks, increased ROS production (generated during the conversion of xanthine dehydrogenase to xanthine oxidase), more injured ECs, and an inability to defend against oxidative stress (Herrick and Matucci Cerinic, 2001; Chung et al., 2006; Ogawa et al., 2006; Servettaz et al., 2009). In line with these observations, increased levels of isoprostanes (oxidized lipids and markers of oxidative stress) have been found to correlate with the extent of vascular damage in SSc (Ogawa et al., 2006, 2010).

Although inhibition of EC growth has previously been found to be associated with NO overproduction, in SSc, a paradoxical decrease in NO bioavailability has been observed (Matucci Cerinic and Kahaleh, 2002). This can partially be explained by the rapid reaction of NO^•^ with the superoxide anion ${\text{O}}_{2}^{•-}$, forming peroxynitrite (Sambo et al., 2001b). With disease progression, the production of eNOS is further downregulated (Bruckdorfer, 2005). As a result of EC injury and subsequent vascular remodeling, SSc patients show specific capillaroscopic patterns consisting of micro-hemorrhages, as well as dilated, giant, and malformed capillaries (Maricq et al., 1980). These nailfold patterns are positively associated with the degree of vasospasm and ischemia, further indicating that ROS plays a role in the occurrence of microvascular injury (van Roon et al., 2016).

### Link Between Oxidative Stress and Auto-immunity

Several SSc-specific auto-antibodies (e.g., anti-topoisomerase 1, anti-centromere antibodies, anti-RNA polymerase III, anti-U3-RNP, TH/To RNP, U1-RNP, and PM-Scl) have previously been identified, and ROS are thought to be involved in the process leading to autoimmunity. More recently, subcutaneous injections of agents generating OH^•^ or HOCl (another ROS) in mice induced cutaneous and lung fibrosis, as well as the production of serum anti-DNA topoisomerase 1 antibodies (Servettaz et al., 2009). Furthermore, it has been shown that injections of ONOO^−^–generating agents induce the production of anti-centromere B antibodies in the absence of serum anti-DNA topoisomerase 1 antibodies. These findings support the hypothesis that bursts of ROS may cause the oxidation of DNA-topoisomerase-1, which may lead to immunological intolerance to this nuclear antigen. In line with these findings, mice exposed to HOCl developed anti-DNA topoisomerase I antibodies and diffuse cutaneous SSc with pulmonary fibrosis (Kavian et al., 2010).

Autoimmunity may occur through the impairment of DNA repair mechanisms (**Figure 2**) (Birben et al., 2012). For example, it has previously been established that 8-oxo-7 hydrodeoxyguanosine levels in lymphocytes were increased in several rheumatic diseases, as compared to healthy controls (Bashir et al., 1993). More recently, autoantibodies against methionine sulfoxide reductase A, one of the antioxidant repair enzymes, have been detected in 33% of SSc patients (Ogawa et al., 2010). This finding supports the hypothesis that an altered defense mechanism may contribute to the development of vasculopathy. In addition, this finding is also indicative of a promutagenic DNA lesion, presumably induced by ROS and defective DNA repair mechanisms (Bashir et al., 1993).

Recently, several studies have investigated the role of a new class of antibodies that may be directly linked to the pathological processes that occur even in the early stages of SSc. For instance, antiendothelial cell antibodies (AECAs) are thought to play an important role in EC injury promoting the development of vasculopathy (Hill et al., 1996; Bordron et al., 1998; Boin and Rosen, 2007; Mihai and Tervaert, 2010). Anti-endothelial cell antibodies may induce endothelial perturbation, an increase in adhesion molecule expression (ICAM-1, VCAM-1, and E-selectin) and stimulate the secretion of pro-inflammatory cytokines and chemokines (Corallo et al., 2015). The formation of AECAs is thought to be initiated by the exposure of the nuclear contents of damaged ECs (Praprotnik et al., 2001) which may very well be induced by excessive ROS production. Moreover, some evidence provided using a molecular cloning strategy suggests that these antibodies (e.g., AECAs) cause oxidative imbalance and subsequently lead to endothelial apoptosis, suggesting causal involvement in the disease process (Margutti et al., 2012).

Some evidence supports the hypothesis that heat shock proteins (HSP) play an important role in the pathogenesis of SSc (Danieli et al., 1992; Ogawa et al., 2008). Heat shock proteins are a family of immunogenic proteins that are found in all organism cells, and are essential for various cellular function. These proteins have chaperon properties and facilitate in protein folding, assembly, and intracellular transport (Brenu et al., 2013). The synthesis of HSP is increased in response to a variety of stressful stimuli, such as hyperthermia, hypoxia, inflammation, and auto-immunity (Kiang and Tsokos, 1998; Fujimoto et al., 2004). Heat shock proteins have previously been found to stimulate antigen presenting cells, which then activate the adaptive immune cells, suggesting a critical role of HSPs in the immune system (Brenu et al., 2013). Furthermore, increased levels of antibodies to HSP have previously been demonstrated in several immune diseases, including atherosclerosis, type 1 diabetes mellitus and various auto-immune rheumatic diseases (Raska and Weigl, 2005; Shukla and Pitha, 2012). Moreover, the 60-kDa heat shock protein (HSP60) was previously found to be a target antigen for AECAs, and thereby induce apoptosis of ECs in variety of rheumatic diseases, including SSc. In support, serum HSP70 levels are also found to be increased in SSc patients, and are associated with the occurrence of fibrosis, oxidative stress, and inflammation (Ogawa et al., 2008). Moreover, expression of HSP47 was found to be linked to the overproduction of type I collagen in cultured SSc fibroblast (Kuroda et al., 1998). These results shed light on the potential of these markers as serological biomarkers of cellular stress. Conversely, there is some evidence supporting the postulation that HSPs also have a beneficial effect following oxidation (Kalmar and Greensmith, 2009). These effects are likely linked to the sensor ability of redox changes and cytoprotective effects of HSPs. However, the exact role in the pathogenesis of SSc has to be elucidated further.

Age related auto-immunity is also thought to play an important role in SSc. Approximately, 21% of all SSc patients are aged 65 years or older at diagnosis (Pérez-Bocanegra et al., 2010). This observation has instigated great interest in the role of key determinants of biological aging (e.g., telomere attrition, senescent cells) in the pathogenesis of SSc. Autoimmunity in the elderly population is also believed to be promoted by accelerated cellular senescence (Piera-Velazquez and Jimenez, 2014) and through the increased affinity of T-cells to self-antigens (Goronzy and Weyand, 2012). It was previously demonstrated that the age of disease onset strongly influences clinical signs of SSc, the presence organ involvement and outcome. Moreover, the elderly SSc patients, compared to their younger counterparts, were more frequently diagnosed with cardiopulmonary organ involvement (Alba et al., 2014). A study conducted by Shiels et al. (2011) demonstrated that patients diagnosed with dcSSc exhibit features consistent with accelerated biological aging, and this was found to be independent of increasing age. This study concluded that abnormal telomere biology may induce the development clinical signs in SSc patients. Supporting this hypothesis, a previously conducted study found that the telomere length of SSc patients, and their family members, were on average 3 Kb shorter than age-matched controls. In addition, they stated that the telomeric length in SSc patients did not decrease significantly with age (Artlett et al., 1996). This reduction in telomere length can partially be explained by the elevated oxidative stress levels in these patients, which may accelerate telomere erosion (MacIntyre et al., 2008). Moreover, this observation may also be explained by the presence of autoantibodies against telomere binding proteins.

### Link Between Oxidative Stress and Inflammation

Chronic ROS overproduction may stimulate a pro-inflammatory state in SSc (Yamagishi et al., 1994; Suzuki et al., 1999). The early phase of SSc is characterized by perivascular mononuclear cell infiltration (Mostmans et al., 2017) and the activation of the innate and adaptive immune system, which results in B-cell and T-cell activation (Amico et al., 2015). These cells secrete pro-inflammatory cytokines (including IL-1, IL-6, and IL-8) and chemokines that further stimulate ROS production (Murrell, 1993; Hua-Huy and Dinh-Xuan, 2015). However, ROS have been reported to promote activation of a variety of inflammation-associated transcription factors (e.g., nuclear factor-kappa B [NF-κB], activator protein-1 [AP-1], p53, signal transducer and activator of transcriptions 3 [STAT3], HIF-1α, and NF-E2 related factor-2 [Nrf2]). This process can further aggravate the inflammatory response through the production of a variety of inflammatory mediators (Hussain and Harris, 2007). Although these findings emphasize that the development of SSc vasculopathy is due to interconnected pathways of both inflammatory processes, as well as oxidative stress, the level of oxidative stress in SSc seems to exceed that which can be explained solely by the inflammatory process. Therefore, our hypothesis that oxidative stress initiates the development of vasculopathy appears to be reasonable.

### Link Between Oxidative Stress and Vascular Wall Remodeling

**FIGURE 4:**
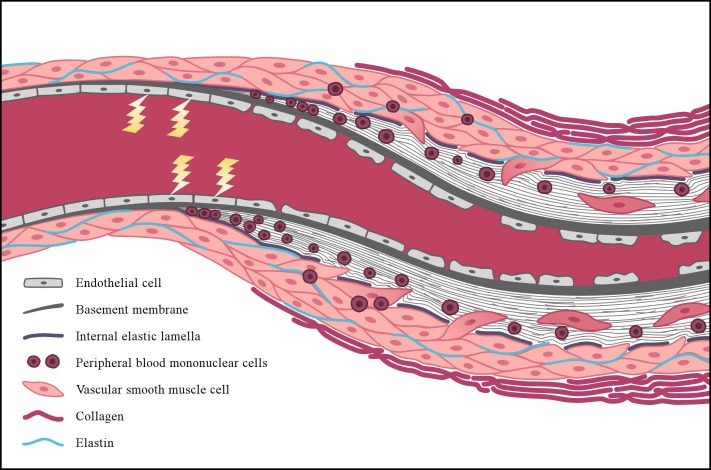
Simplified representation of vascular wall remodeling, leading to critical luminal narrowing of a small vessel as can be seen in patients with SSc. The early events in the pathogenesis of SSc are characterized by endothelial cell (EC) injury, and the lightning bolt represents the initiating factors causing this injury. Reactive oxygen species may play an important initiating role in this process. Following EC injury, EC apoptosis and loss of integrity of the endothelial lining may consequently occur. With the progression of the disease, basement membrane (**continuous gray bold line**) thickening and disruption of the internal elastic lamella are also observed (**dashed line**). Concomitantly, peripheral-blood mononuclear cells (**round nuclear cells**) and vascular smooth muscle cells (VSMCs) (**pink oval-shaped cells**) infiltrate the intimal layer. This process initiates the formation of intimal fibrosis through differentiation of the VSMCs into myofibroblasts, which produce an increased amount of extracellular matrix proteins. Alongside this process, adventitial fibrosis is formed due to increased production of densely packed collagen bundles (**pink bold lines**).

**FIGURE 5:**
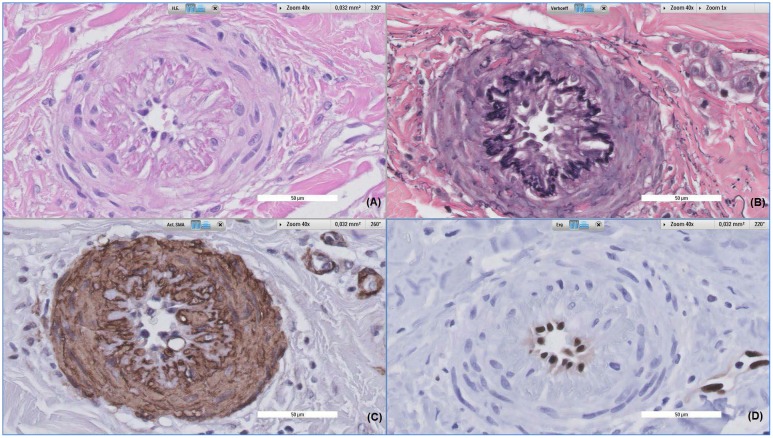
Histopathological alterations of a dermal vessel. Patient, a 62-year-old female, was referred to the dermatologist due to a rash of the lower limbs. All images were taken at 40x magnification, **(A)** shows Hematoxylin and eosin stained tissue, **(B)** shows Verhoeff stained tissue, **(C)** shows alpha-smooth muscle actin stained tissue and **(D)** shows ERG stained tissue. The vessel displayed above shows prominent fibroproliferative alterations, leading to intima and media proliferation; subsequently causing severe narrowing of the vessel lumen and thickening of vessel walls **(A)**. Also, disruption of the internal elastic lamella can be observed **(B)**, with media cells invading the intimal layer **(C)**. This process is accompanied by slight perivascular infiltration of inflammatory cells.

Vascular remodeling affects a variety of sites, including the lungs, the heart, the skin and the kidneys, resulting in critical luminal narrowing of the vessel (**Figures 4**, **5**). Severe complications, such as pulmonary arterial hypertension (PAH) and scleroderma renal crisis (SRC), are accompanied by this luminal narrowing. The occlusion of the microvasculature due to intimal and media proliferation may result in an impaired tissue supply of oxygen and nutrients, leading to increased ROS production and, eventually, to a loss of vasculature (Fleming et al., 2009). In addition, several signals involved in SSc pathogenesis, including transforming growth factor-beta (TGF-β), PDGF, and ET-1 modulate the expression of NOX, which may lead to the formation of fibrosis by up-regulating ROS production (Eckes et al., 2014).

Reactive oxygen species have been implicated in several SSc-related processes, including the stimulation of fibroblast proliferation, collagen-gene expression, and phenotype conversion of myofibroblast (Sambo et al., 2001b). An imbalance between collagen and elastin and the infiltration of the vessel wall by VSMCs, mononuclear cells, and other inflammatory cells, as well as an increased production of cytokines, MMP, chemokines (MCP-1), cell adhesion molecules, and growth factors (TGF-β, CTGF, PDGF), all contribute to vascular wall thickening. Accordingly, it has been observed that extracellular matrix deposition into the vessel wall results from disproportionate fibroblast activity with increased serum levels of hyaluronan, matrix metalloproteinases (MMPs), and tissue inhibitors of metalloproteinases (TIMPs) (Montagnana et al., 2007). TGF-β, in particular, promotes the synthesis of collagen, glycosaminoglycans, and fibronectin by fibroblast, and reduces the collagenase-synthesizing capacity of fibroblasts (Smith and LeRoy, 1990).

Perivascular cells including, but not limited to, smooth muscle cells (SMC), and in particular VSMCs, are thought to play a key role in maintaining vascular integrity. During physiological processes, these cells have a contractile function, and thereby regulate blood flow. Current literature suggest that intimal hyperplasia may be formed by migration and proliferation of medial SMC and adventitial fibroblast (Fleming et al., 2009) as a response to various stimuli including oxidative stress. Subsequently, VSMCs produce excessive amount of ECM to form a fibrotic vascular lesion. Moreover, following the initial vascular injury, endothelial progenitor cells are mobilized from the bone marrow, recruited to the site of the vascular lesion and differentiate into a variety of cells, including VSMCs (Dimmeler et al., 2005). Proliferation of VSMCs is usually regulated by NO, which inhibits cell proliferation through upregulation of cGMP. However, due to the impaired NO production in SSc, processes related to the inhibition of cell proliferation may be altered, and ECM production may be promoted further. In addition to the decreased production of NO, cell proliferation is also promoted by upregulation of ET_B_ receptor on SMCs (Shetty et al., 1993). In addition, migration and proliferation of VSMCs is also likely due to the impaired interaction between ECs and VSMCs. This impaired interaction, which is characterized by a decreased expression of α-SMA in VSMCs, is thought to induce phenotypic modulation in VSMC. This phenotypic modulation induces VSMCs to act in a more in more migratory and proliferative manner (Asano et al., 2010).

Vascular remodeling is also promoted by the ability of ECs to convert into myofibroblast under hypoxic and pro-oxidative milieu, a process known as “endothelial to mesenchymal transition” (Jimenez, 2013). On the other hand, NO has been reported to have an antifibrotic effect in experimental animal models (Yoshimura et al., 2006). NO can directly activate several transcription factors (such as nuclear factor-κB, specificity protein-1, and activator protein) to inhibit collagen gene expression (Bogdan, 2001). In addition, NO mediates prolyl hydroxylase expression, which is important in the post-translational processing of collagen (Dooley et al., 2012). Therefore, the formation of fibrosis may be stimulated by the decreased bioavailability of NO in SSc patients.

HIF-1α is generally increased substantially as a response to low oxygen concentrations, which generally promotes ROS production. This upregulation of HIF-1 controls the expression of several genes involved in processes such as erythropoiesis, angiogenesis, glucose metabolism, cell proliferation, and cell apoptosis (Maxwell and Ratcliffe, 2002; Beyer et al., 2009). Tissue hypoxia usually triggers angiogenesis in order to improve oxygenation of hypoxic tissue. As a response to chronic hypoxia due to progressive loss of vasculature, levels of VEGF often increase. Accordingly, both receptors for VEGF (e.g., VEGF receptors 1 and 2) have previously been found to be overexpressed in the skin and serum of SSc patients (Chora et al., 2017). Reactive oxygen species have been implicated to stimulate the induction of VEGF expression in variety of cells, including ECs, VSMC, and macrophages (Ruef et al., 1997; Wang et al., 2011; Kim and Byzova, 2014). Simultaneously, VEGF further increases the production of ROS through the activation of NADPH oxidase in ECs (Ushio-Fukai and Alexander, 2004).

## Biomarkers of Oxidative Stress

In the past decade, several *in vitro* and *in vivo* studies have demonstrated the role played by the overproduction of free radicals in the pathophysiology of SSc, and several biomarkers of this process have been described. These biomarkers include oxidized low-density lipoprotein, malondialdehyde (MDA), isoprostanes, and circulating total free thiols. Free thiol groups (i.e., −SH) can be oxidized by ROS and other reactive species and are active components of the antioxidant buffer capacity, indicating that their extracellular level can be interpreted as a direct reflection of the overall redox status (Banne et al., 2003; Turell et al., 2013; Cortese-Krott et al., 2017). Lau et al. (1992b) were the first to observe that plasma thiol concentration was reduced in patients with SSc. More recently, we have demonstrated that patients with SSc show decreased levels of free thiols, indicating high oxidative stress. Interestingly, levels of free thiols clearly increased following a cooling experiment of the lower forearm, simulating a Raynaud’s attack (van Roon et al., 2017). Following their initial observation, Lau et al. (1992a) also reported increased levels of MDA, which is a measure of lipid peroxidation. Additionally, increased urinary levels of 8-isoprostanes were previously found in SSc patients in the early stages of the disease (Cracowski et al., 2001). It has also been reported that 8-isoprostane in exhaled breath condensate of SSc patients is increased (Tufvesson et al., 2010). Similarly, it was demonstrated that bronchoalveolar lavage fuid from SSc patients with fibrosing alveolitis contained increased levels of 8-isoprostanes (Montuschi et al., 1998). Furthermore, another study showed that the level of 8-isoprostane not only correlates with the severity of pulmonary fibrosis, but also with the extent of renal vascular damage, and immunological abnormalities in SSc (Ogawa et al., 2006). Collectively, these studies have generated increasing interest in the hypothesis that oxidative stress may play a significant role in the pathogenesis of SSc, and subsequently promote vascular injury. However, in order to optimally use these biomarkers, larger studies investigating the value of these markers as predictors of disease (and disease progression) should be performed.

### Interventional Strategies

Clear evidence indicates the crucial role played by oxidative stress in the occurrence of SSc vasculopathy. For instance, it has been hypothesized that in SSc, ECs are unable to endure prolonged oxidative stress, either through a lack of antioxidant defense mechanisms or because these antioxidant mechanisms are compromised (Herrick and Matucci Cerinic, 2001). As extensively described elsewhere (Herrick and Matucci Cerinic, 2001; Grygiel-Górniak and Puszczewicz, 2014) several studies have investigated the effect of antioxidants in SSc patients. These studies all shared the postulation that antioxidant supplements would decrease susceptibility to oxidative stress-induced tissue damage. Despite some promising results, larger trials have been disappointing (Simonini et al., 2000; Herrick and Matucci Cerinic, 2001). For instance, Niwa et al. (1985) reported that three out of three SSc patients benefitted from liposomal-encapsulated SOD injections. However, Correa et al. (2014) demonstrated that, when taken orally (1800 mg/day), free radical scavenger *N*-acetyl-L-cysteine (NAC) showed no vasodilator effect on the microcirculation of the hands after 4 weeks of treatment in SSc patients. Conversely, others have reported on the beneficial effect of NAC in SSc patients. For example, NAC has been found to diminish cellular ROS in fibroblast and replenish free cellular thiols (Sambo et al., 2001a,b; Servettaz et al., 2007). In addition, an *in vitro* study, conducted in SSc patients, showed that NAC inhibits fibroblast proliferation and collagen synthesis and reduces the formation of peroxynitrite (ONOO^−^) by activated lung macrophages (Ammendola et al., 2002). Moreover, Rosato et al. (2009) demonstrated that treatment with NAC led to a reduction in the number of digital ulcers and decreased the number of RP attacks.

Denton et al. (1999) conducted a randomized parallel group study in 40 RP patients (20 SSc patients, 15 primary RP patients, and 5 secondary RP patients). These patients were randomized to receive either 500 mg daily of the lipid-lowering antioxidant probucol or 20 mg daily of nifedipine. Those patients treated with probucol were found to have a significant reduction in the frequency and severity of Raynaud’s attacks, as well as a rise in LDL oxidation lag time. In addition, hydrogen sulfide (H_2_S), known for its strong antioxidant and vasodilator capacity, was demonstrated to have beneficial effects on bleomycin-induced pulmonary fibrosis in a mice model (Cao et al., 2014). Moreover, Wang et al. (2016) demonstrtaed that H_2_S could improve SSc-related organ fibrosis through the inhibition of the inflammatory reaction and the reduction of TGF-β1 expression. These data place H_2_S in the spotlight as an effective therapeutic agent in SSc patients with organ involvement. Furthermore, there is some evidence indicating that soluble guanylate cyclase (sGC) may be beneficial in SSc. For instance, large randomized controlled clinical trials conducted with riociguat, which directly stimulates sGC in an NO-independent manner, have shown an increase in mean 6-min walking distance and an improvement of pulmonary vascular resistance and cardiac index (Humbert et al., 2017).

Regarding more conventional therapeutic strategies in SSc, the exploratory study conducted by Erre et al. (2008) suggested that a standard course of iloprost therapy, a potent vasodilator, may reduce oxidative stress in SSc patients. This effect appeared to be more consistent in patients in the early phase of the disease. Furthermore, an experimental study conducted by Rafikova et al. (2013) showed that the ET receptor antagonist bosentan (250 mg/kg/day; days 10–21), which is generally prescribed to reduce the number of new DU, not only prevented an increase in right ventricle peak systolic pressure and right ventricle hypertrophy, but also reduced oxidative stress and protein nitration.

## Future Perspectives

Following Murrell’s hypothesis (Murrell, 1993) several studies have investigated the role played by oxidative stress in the pathogenesis of SSc vasculopathy. These studies have not only substantially improved our understanding of this complex disease, but several findings, as presented in this review, support the hypothesis that oxidative stress contributes to, and may even initiate, the occurrence of vasculopathy. This contrasts with the finding that most therapeutic intervention studies with antioxidants have been disappointing. However, some have proposed that these non-conventional therapeutic strategies may only be beneficial in the early stages of the disease, when less severe damage and perhaps lower levels of ROS occur. However, this acts as a double-edged sword. Although universally recognized diagnostic criteria for very early diagnosis of the disease (VEDOSS criteria) have been introduced, these criteria include patients who already have developed EC damage, vasculopathy, and specific antibodies. The inability to identify patients with Raynaud’s who are at increased risk of developing SSc is the primary challenge. Any therapy initiated later in the course of the disease may only mitigate disease processes and symptoms, without actually modifying the disease course and outcome. We postulate that interventions administered early in the course of the disease, before the occurrence of irreversible fibrosis, may prevent irreversible changes by altering the damaging properties of ROS and thereby changing the disease’s course. Although we are encouraging new trials to investigate the effect of antioxidants on SSc, which should include patients in the early stages of the disease, we emphasize that new trials may only be successful when the underlying pathology is fully clarified.

## Author Contributions

AA, GD, MF, DM, and HvG have analyzed the data from literature and designed the review. AA wrote the first draft of the manuscript. DM, HvG, MF, and GD wrote sections of the manuscript. All authors contributed to manuscript revision, read and approved the submitted version.

## Conflict of Interest Statement

The authors declare that the research was conducted in the absence of any commercial or financial relationships that could be construed as a potential conflict of interest.
